# Multiple HPV infections in female sex workers in Western Kenya: implications for prophylactic vaccines within this sub population

**DOI:** 10.1186/s13027-016-0114-5

**Published:** 2017-01-06

**Authors:** Sonia Menon, Davy van den Broeck, Rodolfo Rossi, Emilomo Ogbe, Hillary Mabeya

**Affiliations:** 1 International Centre for Reproductive health, Department of Obstetrics and Gynaecology, Ghent University, De Pintelaan 185 P3, 9000 Ghent, Belgium; 2 Moi University/Gynocare Fistula Centre, Eldoret, Kenya; 3 AMBIOR (Applied Molecular Biology Research Group), Antwerpen, Belgium; 4 Faculty of Medicine and Health Sciences, Laboratory of Cell Biology & Histology, University of Antwerp, Antwerp, Belgium; 5 CDC Foundation Atlanta, Atlanta, USA

**Keywords:** FSW, HIV, High risk HPV, Potential high risk HPV, Multiple pHR/HR coinfections, Vaccine efficacy

## Abstract

**Background:**

Whilst the imputed role of High Risk (HR) HPV infection in the development of cervical lesions and cancer has been established, the high number of HPV genotypes that Female Sex workers (FSW) harbour warrants that the synergistic effects of potential HR (pHR) and HR HPV genotypes be elucidated to assess the potential impact of prophylactic vaccines. This population in Kenya also harbours a number of other vaginal infections and STIs, including bacterial vaginosis (BV), trichomonas vaginalis (TV) and *candida spp*.

The aims of this cross-sectional analysis in Kenya are to explore the epidemiology of abnormal cytology and the pairing of pHR/HPV genotypes in HIV-negative and HIV-infected FSW.

**Methods:**

A cross-sectional study design of 616 FSW from Western Kenya aged between 18 and 61 years during 2009–2015 using a peer recruitment sampling strategy.

**Results:**

Of the 599 FSW who underwent cytological examination, 87 had abnormal cytology (14.5%; 95% CI: 12.0–17.6%). A combined prevalence of HPV16 and 18 (29.6%; 95% CI: 22.2–37.8%) was observed in abnormal cytology. HPV 53 and 51 were the most observed pairing in FSW with abnormal cytology. Significant adjusted associations were found between abnormal cytology and TV (aOR: 30; 95% CI: 14.1–62.9), multiple HR HPV (aOR: 3.7; 95% CI: 1.9–7.3), HPV 51 (aOR 3.7; 95% CI 1.6–8.6) and HPV 52 (aOR 6.1; 95% CI: 2.8–13.3).

**Conclusion:**

HPV 51 and 52 were independently associated with abnormal cervical cytology in both HIV negative/positive FSW. The strong association between TV and cervical dysplasia and the high percentage of FSW harbouring more than one STI underscore the need for enhanced STI management within the framework of cervical cancer prevention.

## Background

Human Papilloma Viruses (HPV) are double-stranded DNA viruses which are now deemed to be the chief etiological agents in cervical intraepithelial neoplasias and cancers [1]. High risk (HR) genotypes, which are associated with cervical cancer include HPV genotypes 16, 18, 31, 33, 35, 39, 45, 51, 52, 56, 58, 59, 66, 68 while HPV types 26, 53, 67, 70, 73 and 82 are now classified as possible carcinogenic, and low risk (LR) HPV genotypes considered benign include 6, 11, 42, 43, and 44 [2]. The 15 HR oncological viral strains, which have been identified can be broken down into different species: the HPV 16 group (alpha-9) of the alpha-papillomavirus genus (HPV 31, HPV33, HPV 35, HPV 52, and HPV 58) and the HPV 18 group (alpha-7) (HPV39, HPV 45, HPV 59, and HPV 68).

Cervical intraepithelial neoplasia (CIN) can be histologically graded into mild dysplasia (CIN 1), moderate dysplasia (CIN 2), and severe dysplasia to carcinoma in situ (CIN 3). Several studies have reported a robust association between HIV and HPV co-infection and therefore the development of CIN and genital cancer, [3, 4] along with a persistence and recurrence of pre-invasive cervical lesions, CIN 2 or CIN 3 [5].

It is well recognized that among the 14 HR HPV genotypes, HPV 16 and 18 are associated with approximately two thirds of all invasive cervical carcinomas [6]. After HPV16/18, data confirm HPV31/33/35/45/52/58 as the most frequently detected genotypes in Invasive Cervical Cancer (ICC) worldwide [7, 8].

Prophylactic vaccines against HPV 16 and 18 are currently being rolled out across the globe for the prevention of cervical cancer, which is likely to yield a significant impact on the future burden of cervical cancer, particularly in sub Saharan Africa, where screening is scarce. Although the bivalent/quadrivalent vaccines, including the LR HPV 6 and 11 constitute a crucial milestone in cervical cancer prevention in HIV-negative women, epidemiological data available suggest that in HIV positive populations, HPV 16 has shown to be frequent, but not as predominant as seen in most HIV negative populations [9, 10]. Moreover, HIV immunosuppression has been linked to multiple HPV infection [11, 12]. Concomitant infection with multiple HPV genotypes has been found to be attributable to the inability to clear HPV infections as well as to the reactivation of latent HPV infections; both occurring as a consequence of immune suppression [13, 14].

Also, epidemiological knowledge of pHR HPV types is highly limited, mainly because commercial molecular assays focus on HR HPV genotypes. There is a paucity of data on these genotypes in HIV positive women with abnormal cytology, notwithstanding their potential enhanced role in cervical dysplasia development in HIV positive patients [15, 16].

In Kenya, the Ministry of Public Health and Sanitation has developed a comprehensive cervical cancer prevention strategy, entailing plans for administrating quadrivalent vaccine to preteen girls in the near future. Currently they are running the pilot programme Kituwi in Eastern Kenya and awaiting approval for nationwide rollout and successful global funding [17]. The not yet commercialized nonavalent vaccine in Kenya, containing additional HPV types HPV 6, 11, 16, 18, 31, 33, 45, 52, and 58 antigens may have the ability to prevent 90% of ICC cases worldwide.

In Kenya, as in many parts of sub-Saharan Africa, where the penal code specifically penalizes prostitution [18], FSW bear the greatest burden of HIV and STI infections. Concomitant STIs and vaginal infections may lead to prolonged HPV infection, which may in turn increase the risk of CIN [19, 20]. Bacterial vaginosis (BV) and *Trichomonas vaginalis* (TV), have been associated with an increased risk of squamous intraepithelial lesions and/or CIN based on biopsy results [21, 22].

As early as 1985, a study reported that HIV prevalence was as high as 61% among a group of FSW in Nairobi [23]. A recent study reported that in Kenya, 5% of the urban female population of reproductive age could be sex workers [24].

The objectives of this analysis were primarily to assess genotype-specific distribution of pHR/HR HPVs in FSW with abnormal cytology, as well as the pairing prevalence of certain pHR/HR HPV genotypes found in HIV negative and HIV infected women with abnormal cytology; secondly, to investigate which HPV genotypes and other variables were associated with abnormal cytology.

## Methods

### Study design

A cross-sectional design was used to explore associations between abnormal cytology and pHR/HR HPV genotypes. This cross-sectional study based on record reviews adhered to the methodological guidelines recommended in the STROBE document on observational studies [25].

Women were excluded if they were pregnant, <18 years of age, had a history of cervical dysplasia or cancer, had current abnormal bleeding or bloody discharge, and/or had a hysterectomy. Snowball sampling was undertaken instead of randomized sampling, which is an often used strategy for locating difficult-to-reach and stigmatized populations [26]. This involved a sample of women engaged in sex work being recruited through informational gatherings, snowball sampling and neighborhood outreach. Inclusion criteria for the study entailed being female, giving consent after being explained the objectives of the study, and having engaged in sex in exchange for money, goods, services, or drugs in the last three months. In order to reduce friendship bias, a limit of referral of 10 FSW was established. This activity was undertaken by means of an extensive community outreach program by Gynocare Women and Fistula Hospital, two non-governmental organizations specializing in reproductive health, to identify women with obstetric fistula, STI screening and cervical screening in Western Kenya. The screening was supported by Gent University, Belgium.

### Sample size

The sample size was calculated to allow for a prevalence of at least 15% for abnormal cytology, [27] with a confidence interval of 95% and a power of 80%.

### Data collection

#### Structured questionnaire

A structured paper questionnaire was privately administered by trained interviewers covering socio-demographic characteristics, and sexual behavior. Participants were offered testing for HIV and HPV. HIV results were disclosed to participants and women infected with HIV received counseling and treatment.

#### Specimen collection and laboratory testing

A gynaecological examination was performed using a swab. Candida colonization was diagnosed by Gram stain; bacterial vaginosis was scored according to Nugent’s criteria. Infection with *Trichomonas vaginalis* was diagnosed by PCR, using a validated method which was part of the HPV genotyping essay.

A HIV diagnosis was performed using rapid immunoassays: Uni-Gold™ Recombigen® HIV (Trinity Biotech plc, Bray, Ireland) and Determine® HIV-1/2 (Abbott Japan co Ltd, Minato-Ku, Tokyo, Japan). In the case of indeterminate results, an enzyme-linked immunosorbent assay was used to confirm HIV status.

#### Biologic specimens

Cervical samples were collected using a cervix brush (Cervex-brush®, Rovers®, Oss, The Netherlands), and cervical cytology was assessed with conventional Papanicolaou (Pap) smears. Slides were read by a cytologist with master level training, supervised by a pathologist. An external cytopathologist provided quality control. The Bethesda Reporting System was used for cytological classification [28].

The cervix brush tips were preserved in a liquid-based cytology collection medium (SurePath®, Tripath Imaging Inc., Burlington, North Carolina, USA) and stored at 4 °C until further processing.

### Ethical approval

Ethical approval for the study was obtained from the Institutional Research and Ethics Committee at the MOI University in Kenya (No 000187) on August 11^th^, 2011.

### Statistics and data analysis

Data analysis was done using STATA version 12 (Stata-Corp LP, College Station, TX). Due to incomplete information about the study samples, we checked whether the missing data (10%) were randomly distributed by performing the Little’s MCAR test. Continuous variables were then converted into categorical. Age was dichotomized into ≥30 years and <30 years; this categorization was used to reflect the WHO 2014 guideline concerning cervical screening. The number of pHR/HR HPV co-infections was also dichotomized as a categorical variable with 1 and ≥2 genotypes.

We first described the distribution of pHR/HR HPV types observed among women with both normal cytology and abnormal cytology, for which the overall prevalence and 95% confidence intervals (95% CI) based upon normal distributions were calculated.

To examine patterns of clustering of high-risk HPV types, the prevalence of pHR/HR HPV genotypes in presence of another pHR/HR HPV genotypes by abnormal cytology was calculated, which was defined as the proportion of women with abnormal cytology who were positive for the pHR/HR HPV genotypes. The prevalence of pairings detected in women with HSIL was also calculated.

The variables were explored by means of tabulation and cross-tabulation. The *χ* ^2^ test was used to assess whether there was an association between CIN 2+ and various risk factors. In building the regression models, age, STIs and multiple pHR/HR HPV genotypes tested were entered. A multivariable logistic regression analysis was performed to assess the association between pHR/HR HPV genotypes and abnormal cytology, ASC-US or higher, the main outcome of interest and to simultaneously control for potential confounders. The Likelihood Ratio Test (LRT) was used to measure the association of each variable with the outcome.

To assess for a possible interaction due to age, logistic regression models were fitted with and without the interaction term; significance for interaction was then checked through visual inspection of the OR and LRT. Statistical significance was considered at *p* ≤ 0.05.

## Results

Out of the 616 participants, data from only 599 participants could be analysed because of the quality of the specimen. Missing values were observed for BV (61), TV (7) and *candida spp* (7). The Little’s MCAR test revealed that the missing values were randomly distributed among the participants (*p* = 0.4), therefore unlikely to have introduced information bias.

The mean age of study participants was 28 years and median parity was 2 (range: 0–7 children). Regular condom use, which was defined as always or almost always was reported in 21.7% of 369 women. (95% CI = 17.6–26.2%). The median number of partners in the past week was 4 (IQR: 2–7). Table 1 depicts the prevalence of categories for age and sexual behavior.

**Table 1:** Reports the prevalence of categories for age and sexual behaviour

Socio demographic variables	Percentage (95% CI)
>30 years	40.1% (95% CI: 36.2–44.1)
≤30 years	59.9% (95% CI: 55.9–63.8)
Sexual behavior:	
> 4 sexual partners the past week	1.9% (95% CI:1.0–3.3)
≤ 4sexual partners	98.1% (95% CI: 96.7–99.0)
Regular use of condom	21.7% (95% CI 17.6–26.2)
No regular use of condom	78.3% (95% CI: 73.8–92.3)

Of the 616 FSW who underwent cytological examination, 512 (85.5%: 95% CI: 82.4–88.2) had normal cytology, 87 had abnormal cytology (14.5%; 95% CI: 12.0–17.6%) and 17 were excluded due to poor quality of the sample, leaving 599 participants on whom we could perform the analysis. Of the FSW population, 192 FSW were HIV positive, of which 27.1% (95% CI: 20.9–34.0%) had abnormal cytology. Table 2 in annex reports the prevalence of cervical abnormalities observed in the sample (*N* = 616).

**Table 2:** Reports the prevalence of cervical abnormalities observed in the sample (*N* = 616)

Cytological status	n	% of FSW (95% CI)
Normal cytology	512	85.5% (82.4–88.2)
ASC-US	10	1.7% (0.8–3.04)
LSIL	63	10.5% (8.2–13.3)
HSIL	14	2.3% (12.8–3.9)
Excluded samples due to poor quality	17	2.8%

The prevalence of pHR/HR HPV and multiple pHR/HR co-infections in the 616 FSW was 57.7 and 32.8% respectively. In HPV infected FSW, HIV-infected FSW had a significantly higher number of co-infections (2.0) than HIV negative FSW (0.9), *p* < 0.001.

The prevalence of BV in this population was 48.3%, followed by TV 31.4%, and *candida spp* 19.9%. FSW with BV had the highest prevalence of multiple pHR/HR co-infections with 53.4%, followed by TV, and *candida spp*, 38.8and 23.9% respectively. Table 3 reports the prevalence of each HPV genotypes and vaginal infections/TV.

**Table 3:** Reports the prevalence of each HPV genotypes and vaginal infections/TV

pHR/HR HPV Genotype	Frequency (n)	Percentage (*N* = 616)	95% CI
HPV 16 (*N* = 616)	99	16.10%	13.3%–19.2%
HPV 18 (*N* = 616)	68	11.04%	08.7%–13.8%
HPV 31 (*N* = 616)	49	8.00%	5.9%–10.4%
HPV 33 (*N* = 616)	2	0.30%	0.04%–1.2%
HPV 35 (*N* = 616)	70	11.40%	9.0%–14.1%
HPV 39 (*N* = 616)	48	7.80%	5.8%–10.2%
HPV 51 (*N* = 615)	52	8.50%	3.7%–7.4%
HPV 53 (*N* = 616)	68	11.04%	8.7%–13.8%
HPV 56 (*N* = 616)	45	7.30%	5.4%–9.7%
HPV 58 (*N* = 616)	30	4.90%	3.3%–6.9%
HPV 59 (*N* = 616)	75	12.20%	9.7%–15.02%
HPV 66 (*N* = 616)	60	9.70%	7.5%–12.4%
HPV 68 (*N* = 616)	9	4.90%	3.3%–6.9%
Vaginal infections and TV			
BV (*N* = 555)	268	48.30%	44.06%–52.5%
TV (*N* = 609)	191	31.4%	27.6%–35.2%
Candida (*N* = 609)	121	19.90%	16.8%–23.3%

### Normal and abnormal cytology and each HPV genotypes

The combined prevalence of HPV 16 and HPV 18 was 27.1%, and of the two pHR HPV genotypes tested, HPV 53 and 66, 20.8% (95% CI: 17.6–24.2%). In women with abnormal cytology, we observed a multiple pHR/HR HPV genotype prevalence of 65.5%. See Table 4 in annex, which reports the prevalence of each HPV genotypes.

**Table 4:** Reports the prevalence of each HPV genotype in women with abnormal cytology

HPV genotype	Abnormal cytology	% (*N* = 87)	95% CI
HPV 16	24	28.6%	19.2–39.5
HPV 18	15	17.9%	10.0–27.0
HPV 31	13	15.5%	8.5–25.0
HPV 33	1	1.2%	0.03–6.5
HPV 35	18	21.4%	13.2–31.7
HPV 39	13	15.5%	8.5–25.0
HPV 45	9	10.7%	5.0–19.4
HPV 51	18	21.4%	13.2–31.7
HPV 52	26	31.0%	21.3–42.0
HPV 53	21	25.0%	16.2–35.6
HPV 56	13	15.5%	8.5–25.0
HPV 58	4	13.3%	3.8–30.7
HPV 59	17	23.6%	14.4–35.1
HPV 66	9	10.7%	5.0–19.4
HPV 67			
HPV 68	4	4.8%	1.3–11.7

### Potential HR HPV in abnormal cytology

HPV 53 and HPV 66 were found as a stand-alone pHR HPV genotype in two LSIL cases.

### Pairings of pHR/HR HPV genotypes in abnormal cytology

A higher prevalence of pairings in abnormal cytology were observed in HIV infected FSW than HIV negative FSW. The most frequently observed pairings in HIV- negative FSW were HPV 18 and 31 (*n* = 3) occurrences, and HPV 31 and 52 (*n* = 2), involving genotypes phylogenetically related to the HPV 16.

In HIV-infected women, HPV 53 and HPV 51 are observed in 5 of the most prevalent pairings, followed by HPV 16, observed in 4 of the most prevalent pairings. Whilst in HIV negative FSW, the most prevalent co-infection pairings exhibited a mixed alpha 9-alpha 7 combination or a homogenous alpha 9 pattern, in HIV infected women, the non-alpha 9/7 pHR HPV genotypes, HPV 53 (alpha 6) and HPV 51 (alpha 5) figure prominently. See Table 5 in annex which depicts the most prevalent pairing occurrences in women with abnormal cytology and HSIL.

**Table 5:** Most prevalent pairing occurrences in women with abnormal cytology and HSIL

Prevalent pairings in abnormal cytology in HIV-negative women	Occurrences (n)	% in abnormal cytology
HPV 18 and 31	2	3
HPV 31 and 52	7	2
Prevalent pairings in HIV infected women with abnormal cytology		
HPV 16 and 39	2	6
HPV 16 and 52	9	7
HPV 16 and 51	4	5
HPV 16 and 53	10	7
HPV 18 and 52	12	5
HPV 18 and 53	8	5
HPV 31 and 51	2	5
HPV 35 and 51	2	5
HPV 35 and 53	4	7
HPV 45 and 53	0	6
HPV 45 and 59	2	5
HPV 51 and 53	2	7
HPV 51 and 56	1	6
HPV 52 and 56	3	6
HPV 53 and 56	1	5

### Risk factors for abnormal cytology

In the univariate analysis, age did not appear to be associated with abnormal cytology: women under 30 years of age have a crude OR 1.1 (95% CI: 0.7–1.8 *p* = 0.6) of having abnormal cytology compared to older women.

A very strong significant association was found between TV and abnormal cytology OR: 30.0 (95% CI: 14.1–62.9) adjusting for age and pHR/HR HPV genotypes, BV and *candida spp* and HIV.

A statistically significant OR, adjusted for age was found for multiple HPV and abnormal cytology compared to single HPV genotype infection. This association decreased but remained significant (OR; 3.9; *p* < 0.001; 95%CI: 1.9–7.8) when adjusted for HIV.

Whilst all pHR/HR HPV genotypes were significant predictors of abnormal cytology, when adjusted for age pHR/HR HPV genotypes co-infections, HIV, BV, TV, and *candida spp* these associations became statistically insignificant, except for HPV 51 and 52. Table 6 in annex depicts the age, BV, TV and *candida sp*p adjusted association between specific pHR/HR HPV genotypes and abnormal cytology.

**Table 6:** Association between vaginal infections, TV, specific pHR/HR HPV genotypes and abnormal cytology; OR from Logistic regression; *p*-value from LRT

Vaginal infections and STIs	OR Model 1 (95% CI)	*p*-value	OR Model 2 (95% CI)	*p*-value
BV	0.9 (0.6–1.5)	0.8	0.8 (0.5–1.4)	0.5
TV	24.8 (12.7–48.3)	<0.001	30.0 (14.1–62.9)	<0.001
Candida spp	1.0 (0.5–1.7)	1.0	0.9 (0.5–1.7)	0.7
Multiple HPV infection	5.3 (2.9–9.7)	<0.001	3.7 (1.9–7.3)	<0.001
HPV 16	1.9 (0.8–4.5)	0.1	1.2 (0.5–3.2)	0.5
HPV 18	0.8 (0.3–2.1)	0.7	1.04 (0.4–2.8)	0.9
HPV 31	0.5 (0.2–1.5)	0.2	0.6 (0.2–1.7)	0.3
HPV 33	3.9 (0.05–293.9)	0.5	2.8 (0.03–254.6)	0.6
HPV 35	1.3 (0.6–3.0)	0.5	1.1 (0.5–2.7)	0.9
HPV 39	3.3 (1.3–8.7)	0.03	2.5 (0.9–7.1)	0.09
HPV 51	3.7 (1.6–8.6)	0.002	3.7 (1.5–9.0)	0.004
HPV 52	6.1 (2.8–13.3)	<0.001	4.0 (1.6–8.2)	0.002
HPV 53	2.0 (0.8–4.9)	0.1	1.4; (0.5–3.8)	0.5
HPV 56	2.5 (1.0–6.6)	0.06	2.0 (0.7–5.7)	0.2
HPV 58	0.9 (0.2–3.6)	0.9	1.1 (0.3–5.2)	0.9
HPV 66	1.2 (0.5–3.0)	0.7	1.0 (0.4–3.0)	0.9
HPV 68	1.7 (0.2–17.0)	0.7	0.8 (0.1–7.4)	0.8

Model 1: OR adjusting for age, pHR/HR HPV genotypes, vaginal infections/TV

Model 2: OR adjusting for age and pHR/HR genotypes, vaginal infections/TV and HIV

As no interaction terms were significant, no result reflecting the differential impact for that particular outcome was presented.

## Discussion

### Summary of results

In the present study, we observed a high prevalence of women harbouring more than two pHR/HR HPV genotypes, which was significantly higher in HIV-1–infected women, consistent with results of several studies illustrating that HIV-1–infected women not only have a greater prevalence of HR-HPV infection but multiple coinfections [29, 30].

Our observations are in agreement with those of a large study on multiple HPV infections in Costa Rica in which young healthy women with multiple infections were at significantly increased risk of CIN 2+, Although our findings of an association between TV and abnormal cytology are congruent with other observations [31] demonstrating an association between TV and abnormal cytology, [32, 33] our study suggest an unprecedented strong association.

After adjusting for age and multiple pHR/HR HPV co-infections, BV, TV and *candida spp*, no significant association was observed between any single pHR/HR HPV and abnormal cytology, except for HPV 51 and 52 in both HIV negative and positive women. This is incongruent with our previous findings in an exclusively HIV-infected study population in Belgium, [34] in which only the association between HPV 39 and abnormal cytology became statistically significant.

In contrast to HPV 31 and 58 being the most observed frequent pairing in Brazil in HIV infected women, and HPV 31 and 66 and HPV 39 and 52 in our Belgian study population, in this FSW study population, HPV 31, 52 and 66 do not figure prominently within pairings observed in HIV-infected women despite a higher prevalence of HPV 52 than HPV 53.

Also, our high prevalence of women with abnormal cytology without any detected HPV genotypes can be attributed to our testing of only 18 genotypes out of the over 200 HPV types described. Whilst some types may be inducing low grade lesions, they may also lack a cancer-initiating capacity.

### Strengths and limitations

Our major strength was that all cervical smears were histologically confirmed and the high sensitivity of the HPV DNA diagnostics employed. However, whilst our sample was large, the number of FSW with abnormal cytology was small, which precluded us from exploring particular co-infection patterns as a risk factor. Moreover, due to our lack of behavioral and clinico-epidemiological data, including smoking, CD4 count, HAART use or the presence of other co-infections, we have not been able to adjust for these potential confounders, nor assess whether these factors were associated with cervical abnormalities. Also, there may be potential for selection bias as FSW with similar characteristics may have been sampled, as a result of the convenience sampling method used. However, a convenience sampling strategy may have inadvertently excluded those FSW operating in a more clandestine fashion and thereby not benefitting from a social network.

A limitation related to a cross sectional study design may be the lack of data concerning age of acquisition of HIV infection since it is possible this may have occurred too late in life for some of the women in our study to influence abnormal cytology. Similarly, an analysis of a cross sectional study for exploring associations between multiple HPV genotypes and abnormal cytology may have inherent limitations as infections may have been acquired concurrently or sequentially, therefore, resulting in the criterion of temporality for causation not being met. This may have an impact as immunologic responses may differ following concurrent acquisition of multiple HPV genotypes from infections that are acquired sequentially.

### Implications for vaccination programs

The only significant association observed between abnormal cytology and HPV 51 and HPV 52 underscore the need for post quadrivalent and nonavalent vaccine surveillance in both HIV negative and HIV infected FSW.

In light of a high prevalence of multiple HR HPV infections in this FSW HIV-negative and infected population, it will need to be elucidated whether cross protection may be hampered by the additional burden due to other synergistic relationships among HR HPV genotypes present. A recent systematic review and meta-analysis [35] found that bivalent Cervarix© vaccine from GlaxoSmithKline had better cross protection against HPV 31 in persistent infection, but that efficacy against persistent infections with types 31 appeared to decrease with longer follow-up, suggesting a waning of cross-protection. It still remains to be determined whether a cross protection can be extrapolated to HIV-infected women and in presence of multiple HR HPV genotypes.

Moreover, with a high prevalence of the pHR HPV 53 in pairings with the vaccine preventable HPV 16 and HPV 18 in HIV infected women, it will need to be determined how HPV 53 will fare within the vacuum that ensues the quadrivalent vaccine [36]. In addition, given the high median number of pHR/HR HPV genotypes harboured by this population, the synergies between not only two pHR/HR HPV genotypes need to be determined but within a context of other prevalent genotypes, capable of inducing cervical cancer genesis. Whether Gardasil and Cervarix can attain a 70% reduction of cervical cancer may be contingent upon the natural history of the imputed pHR/HR HPV genotypes in cancer genesis along with their synergistic effects.

Our very high association between TV and cervical dysplasia underscores the need for STI management to be integrated within cervical cancer prevention program. Furthermore, the biological interaction between TV and HPV and its subsequent capacity to induce progression of cervical dysplasia should be further explored. Moreover, the impact of immune modulating infections, such as tuberculosis, malaria and helminthic infections on cervical disease progression should be elucidated in HIV triple co-infected women with HPV and TV.

## Conclusion

Co-infection with pHR/HR HPV genotypes was more strongly associated with abnormal cytology than any single high-risk HPV. In light of the high prevalence of multiple pHR/HR HPV genotypes harboured by FSW and especially HIV infected women, its micro epidemiology in cervical carcinoma in HIV positive women needs to be explored in order for the vaccine efficacy to be assessed.

Whilst the quadrivalent vaccine may be effective in reducing the prevalence of abnormal cytology in HIV negative and HIV infected FSW, the high presence of multiple infections with HPV 16 requires that the micro epidemiology of concurrent be elucidated. In particular, the high prevalence of the non alpha 9 and 7 genotypes, HPV 51 and HPV 53 observed in pairings with HPV 16 and HPV 18 in HIV infected FSW requires further characterization.

These current gaps in epidemiology underscore the need for FSW, HIV negative or positive to be regularly monitored in the post quadrivalent/nonavalent vaccine era.

The strong association observed between TV and cervical dysplasia as well as the high percentage of FSW harbouring more than one vaginal infection/STI begs for the elucidation of synergistic interactions between multiple STIs to be better assessed as factor(s) for cervical dysplasia and for a wider encompassing cervical cancer prevention framework.

## Abbreviations

ASC-H: Typical squamous cells-cannot exclude high-grade squamous intraepithelial lesion
ASC-U: Atypical cells of undetermined significance
FSW: Female sex workers
HIV: Human immunodeficiency virus
HPV: Human Papilloma virus
HSIL: High grade squamous intraepithelial lesion
LR HPV: Low risk HPV
LSIL: Low grade squamous intraepithelial lesion
pHR HPV: Potential high risk
