# Correction to: Multiple HPV infections in female sex workers in Western Kenya: implications for prophylactic vaccines within this sub population

**DOI:** 10.1186/s13027-018-0209-2

**Published:** 2019-01-21

**Authors:** Sonia Menon, Davy van den Broeck, Rodolfo Rossi, Emilomo Ogbe, Hillary Mabeya

**Affiliations:** 1 International Centre for Reproductive health, Department of Obstetrics and Gynaecology, Ghent University, De Pintelaan 185 P3, 9000 Ghent, Belgium; 2 Gynocare Fistula Centre, Moi University, Eldoret, Kenya; 3 AMBIOR (Applied Molecular Biology Research Group), Antwerpen, Belgium; 4 Faculty of Medicine and Health Sciences, Laboratory of Cell Biology & Histology, University of Antwerp, Antwerp, Belgium; 5 CDC Foundation Atlanta, Atlanta, USA

## Correction to: Infectious Agents and Cancer 2017 12:2 DOI 10.1186/s13027-016-0114-5

Following publication of the original article [1], the author reported that some values in Tables 2, 3, 4, 5 and 6. The correct Tables are as given below:

**Table 2:** reports the prevalence of cervical abnormalities observed in the sample (*N* = 599)

Cytological status	n	% of FSW (95%CI)
Normal cytology	512	85.5% (82.4–88.2)
ASC-US	10	1.7% (0.8–3.04)
LSIL	63	10.5% (8.2–13.3)
HSIL	14	2.3% (12.8–3.9)
Excluded samples due to poor cell quality	17	2.8%

**Table 3:** reports the prevalence of each HPV genotypes and STIs

pHR/ HR HPV Genotype	Frequency (n)	Percentage
HPV 16 (*N* = 616)	99	16.1%
HPV 18 (*N* = 616)	68	11.0%
HPV 31 (*N* = 616)	49	8.0%
HPV 33 (*N* = 616)	2	0.3%
HPV 35 (*N* = 616)	70	11.4%
HPV 39 (*N* = 616)	48	7.8%
HPV 51 (*N* = 615)	52	8.5%
HPV 53 (*N* = 616)	68	11.0%
HPV 56 (*N* = 616)	45	7.3%
HPV 58 (*N* = 616)	30	4.9%
HPV 59 (*N* = 616)	75	12.2%
HPV 66 (*N* = 616)	60	9.7%
HPV 68 (*N* = 616)	9	1.5%
BV and STIs		
BV* (*N* = 555)	268	48.3%
TV (*N* = 609)	191	31.4%
Candida (*N* = 609)	121	19.9%

**Table 4:** reports the prevalence of each HPV genotype among HPV-positive women with abnormal cytology

HPV genotype	Abnormal cytology	% (*N* = 84)
HPV 16	24	28.6%
HPV 18	15	17.9%
HPV 31	13	15.5%
HPV 33	1	1.2%
HPV 35	18	21.4%
HPV 39	13	15.5%
HPV 45	9	10.7%
HPV 51	18	21.4%
HPV 52	26	31.0%
HPV 53	21	25.0%
HPV 56	13	15.5%
HPV 58	4	4.8%
HPV 59	17	20.2%
HPV 66	9	10.7%
HPV 68	4	4.8%

**Table 5:** most prevalent pairing occurrences in women with abnormal cytology

Prevalent pairings in abnormal cytology in HIV-negative women: *N* = 35)	(*n* = in normal cytology)	*n* = abnormal cytology
HPV 18 and 31	2	3
HPV 31 and 52	7	2
Prevalent pairings in HIV infected women with abnormal cytology: *N* = 52)		
HPV 16 and 39	2	6
HPV 16 and 52	9	7
HPV 16 and 51	4	5
HPV 16 and 53	10	7
HPV 18 and 52	12	5
HPV 18 and 53	8	5
HPV 31 and 51	2	5
HPV 35 and 51	2	5
HPV 35 and 53	4	7
HPV 45 and 53	0	6
HPV 45 and 59	2	5
HPV 51 and 53	2	7
HPV 51 and 56	1	6
HPV 52 and 56	3	6
HPV 53 and 56	1	5

**Table 6:** association between STI, specific pHR/HR HPV genotypes and abnormal cytology; OR from

STI or HPV genotype	OR Model 1 (95%CI)	*p*-value	OR Model 2 (95%CI)	*p*-value
BV	0.9 (0.6–1.5)	0.8	0.8 (0.5–1.4)	0.5
TV	24.8 (12.7–48.3)	< 0.001	30.0 (14.1–62.9)	< 0.001
Candida spp	1.0 (0.5–1.7)	1.0	0.9 (0.5–1.7)	0.7
Multiple HPV infection	5.3 (2.9–9.7)	< 0.001	3.7 (1.9–7.3)	< 0.001
HPV 16	1.9 (0.8–4.5)	0.1	1.2 (0.5–3.2)	0.5
HPV 18	0.8 (0.3–2.1)	0.7	1.04 (0.4–2.8)	0.9
HPV 31	0.5 (0.2–1.5)	0.2	0.6 (0.2–1.7)	0.3
HPV 33	3.9 (0.05–293.9)	0.5	2.8 (0.03–254.6)	0.6
HPV 35	1.3 (0.6–3.0)	0.5	1.1 (0.5–2.7)	0.9
HPV 39	3.3 (1.3–8.7)	0.03	2.5 (0.9–7.1)	0.09
HPV 51	3.7 (1.6–8.6)	0.002	3.7 (1.5–9.0)	0.004
HPV 52	6.1 (2.8–13.3)	< 0.001	4.0 (1.6–8.2)	0.002
HPV 53	2.0 (0.8–4.9)	0.1	1.4; (0.5–3.8)	0.5
HPV 56	2.5 (1.0–6.6)	0.06	2.0 (0.7–5.7)	0.2
HPV 58	0.9 (0.2–3.6)	0.9	1.1 (0.3–5.2)	0.9
HPV 66	1.2 (0.5–3.0)	0.7	1.0 (0.4–3.0)	0.9
HPV 68	1.7 (0.2–17.0)	0.7	0.8 (0.1–7.4)	0.8

Logistic regression; *p*-value from LRT

Model 1: OR adjusting for age, pHR/HR HPV genotypes, STIs

Model 2: OR adjusting for age and pHR/HR genotypes, STIs and HIV
